# Effects of Tumor Necrosis Factor-β (*TNF-β*) 252A>G Polymorphism on the Development of Migraine: A Meta-Analysis

**DOI:** 10.1371/journal.pone.0100189

**Published:** 2014-06-24

**Authors:** Ruozhuo Liu, Minghui Ma, Mingyu Cui, Zhao Dong, Xiaolin Wang, Wei Zhang, Minghui Yang, Shengyuan Yu

**Affiliations:** 1 Department of Neurology, Chinese PLA General Hospital, Beijing, China; 2 Ministry of Resource Construction, Medical Library of the Chinese PLA, Academy of Military Medical Sciences, Beijing, China; 3 Department of Traditional Chinese Medicine, Chinese PLA General Hospital, Beijing, China; INSERM U894, Centre de Psychiatrie et Neurosciences, Hopital Sainte-Anne and Université Paris 5, France

## Abstract

**Background and Objective:**

Genetic factors including *TNF-β* have been considered as important components in the aetiology of migraine. Many studies have investigated the association between *TNF-β* 252A>G polymorphism and migraine risk, with debatable results. This study was designed to examine whether the *TNF-β* 252A>G polymorphism confers genetic susceptibility to migraine in diverse populations.

**Method:**

Studies eligible for this meta-analysis were searched in the PubMed, Embase, and CNKI by using the keywords “tumor necrosis factor”, “*TNF*”, “252A>G”, “rs909253”, “polymorphism”, “polymorphisms”, “variant”, “SNP”, combined with “migraine” or “migraine with aura (MA)” or “migraine without aura (MO)”. Pooled ORs and 95% CI were appropriately calculated using the fixed-effect model.

**Results:**

We finally included a total of seven studies, providing 5 557 migraineurs and 20 543 unrelated healthy controls. Meta-analysis results showed no statistical evidence of a significant association between *TNF-β* 252A>G polymorphism and overall migraine risk. Stratified analyses by type of migraine and gender revealed similar results. Interestingly, an OR with 95% CI representing an increased migraine risk was indicated in Asians under the recessive model (GG vs. AG + AA: OR, 1.38; 95%CI, 1.04–1.84; P for heterogeneity, 0.665).

**Conclusions:**

Our findings appear to support the hypothesis that genetic variability of 252A>G polymorphism in *TNF* region may modulate risk of migraine in the population of Asian ancestry.

## Introduction

Migraine is a chronic neurological disorder accompanied with digestive system and autonomic nervous system symptoms composed of nausea, vomiting and extreme photophobia, and phonophobia [1], [2]. It is estimated that 8.4% of the general population (10% of men and 24% of women) are affiliated by the common disease [3]–[5]. With the characteristics of severity and intense throbbing pain in the head, migraine has been ranked as one of the top 20 most debilitating diseases worldwide [6]. Despite the currently incomplete definition of the exact pathophysiology of migraine, cytokine-related genes, such as tumor necrosis factor (*TNF*), interleukin-1β (*IL-1β*) and *IL-10*, are known genetic candidates contributing to the predisposition towards migraine development [7]–[9].

*TNF* (*TNF-α* andlymphotoxin-α or *LT-α* or *TNF-β*) are cytokines implicated to influence the intensity and duration of local inflammation. *TNF* could act as an inflammatory mediator in the activation and sensitization of meningeal nociceptors, threshold brain excitability, and propagation of neuronal hyperexcitability, consequently leading to persistent pulsating headache characterized in migraine [10], [11]. Single nucleotide polymorphisms (SNPs) within this locus are likely to be genetically associated with a variety of autoimmune and infectious diseases, due to the major histocompatibility complex and biological activities in the location of this gene [12].

*TNF-β* mapped on chromosome 6 is a member of the *TNF* cytokine superfamily, playing an important role in immune and inflammatory responses [13]. Polymorphisms of this gene have been found to have regulatory function in cytokine levels of *TNF-β* [14]. 252A>G polymorphism (rs909253) is such a variant with silent point mutation capable of modifying gene expression and has been investigated in migraine risk research in multiple populations. The susceptibility of 252A>G polymorphism was primarily reported in a population of Italian ancestry, with a discovery suggesting that carriage of the A allele conferred high risk for the development of migraine without aura (MO) [15]. Since then, several follow-up studies have been continuously carried out in an effort to replicate the initial finding. Some were successful [16], [17], whereas others failed [18], [19]. Multiple factors including selection of diverse populations, different control sources, and misclassification of genotypes may individually or jointly result in the controversy, but the main source can attribute to the various numbers of subjects used in each study.

The aim of this study was to determine whether 252A>G polymorphism in the *TNF-β* region is linked with the risk of migraine. We hypothesized that the 252A>G polymorphism may be associated with migraine risk. The hypothesis was examined though a meta-analysis of 5 557 migraineurs and 20 543 unrelated healthy controls from seven publications.

## Materials and Methods

### Literature source and search strategy

Studies examining the association between *TNF-β* 252A>G polymorphism and migraine were systematically searched using the keywords “tumor necrosis factor”, “*TNF*”, “252A>G”, “rs909253”, “polymorphism”, “polymorphisms”, “variant”, “SNP”, combined with “migraine”, or “migraine with aura (MA)” or “migraine without aura (MO)” in the PubMed, Embase, and China national knowledge infrastructure (CNKI) by two independent investigators. A manual search for potentially relevant studies was also conducted by reviewing the literature identified in the electronic databases. The last search was updated in June, 2013.

### Inclusion criteria

The meta-analysis only included the articles i) that evaluated the association of *TNF-β* 252A>G polymorphism and migraine risk between patients and matched controls; ii) that contained genotype frequency in full detail allowing for calculation of crude risks for migraine. We excluded the studies reporting the association between *TNF-β* 252A>G polymorphism and survival from migraine and/or response to any therapy. If more than one article was published by the same author sharing the same cases series, we selected the study with the most subjects.

### Data extraction

Usable data were independently extracted from each paper by two investigators using a structured sheet and then pooled into a database. The items recorded were first author, publication year, location of the study, racial descent of the studied subjects, type of controls, mean age of the cases, total number of cases and controls for migraine, MA and MO, and the corresponding genotype distributions.

### Statistical analysis

Statistic analysis was performed by using STATA version 12.0 (Stata Corporation, College Station, TX). The significance threshold was defined as P<0.05 unless specially stated. Deviation from Hardy-Weinberg equilibrium (HWE) was tested using the goodness-of-fit χ^2^ test for each control group of the included studies. An odd ratio (OR) with 95% confidence interval (CI) was measured to estimate the association between 252A>G polymorphism and migraine risk. The significance for pooled ORs was evaluated by Z test. The ORs of homozygous model (GG vs. AA), dominant model (GG+AG vs. AA), recessive model (GG vs. AG+AA), allele model (G vs. A), and heterozygous model (AG vs. AA) were calculated by the fixed effects model or the random effects models according to the outcome of between-study heterogeneity detected by the Chi square based Q test [20]. The fixed effects model with the use of Mantel-Haenszel (M–H) method [21] was performed for estimates of the pooled ORs when heterogeneity was indicated to be non-significant (P>0.05); otherwise, the random effects model based on DerSimonian and Laird (D–L) method [22] was more appropriate.

Stratified analyses were performed by racial descent, type of migraine, and gender. The leave-one-out sensitivity analysis was carried out to detect the extent of influence from the single studies on the combined results. The funnel plot and the Egger's test were applied to evaluate publication bias across studies [23].

## Results

### Study inclusion and characteristics

**Figure 1:**
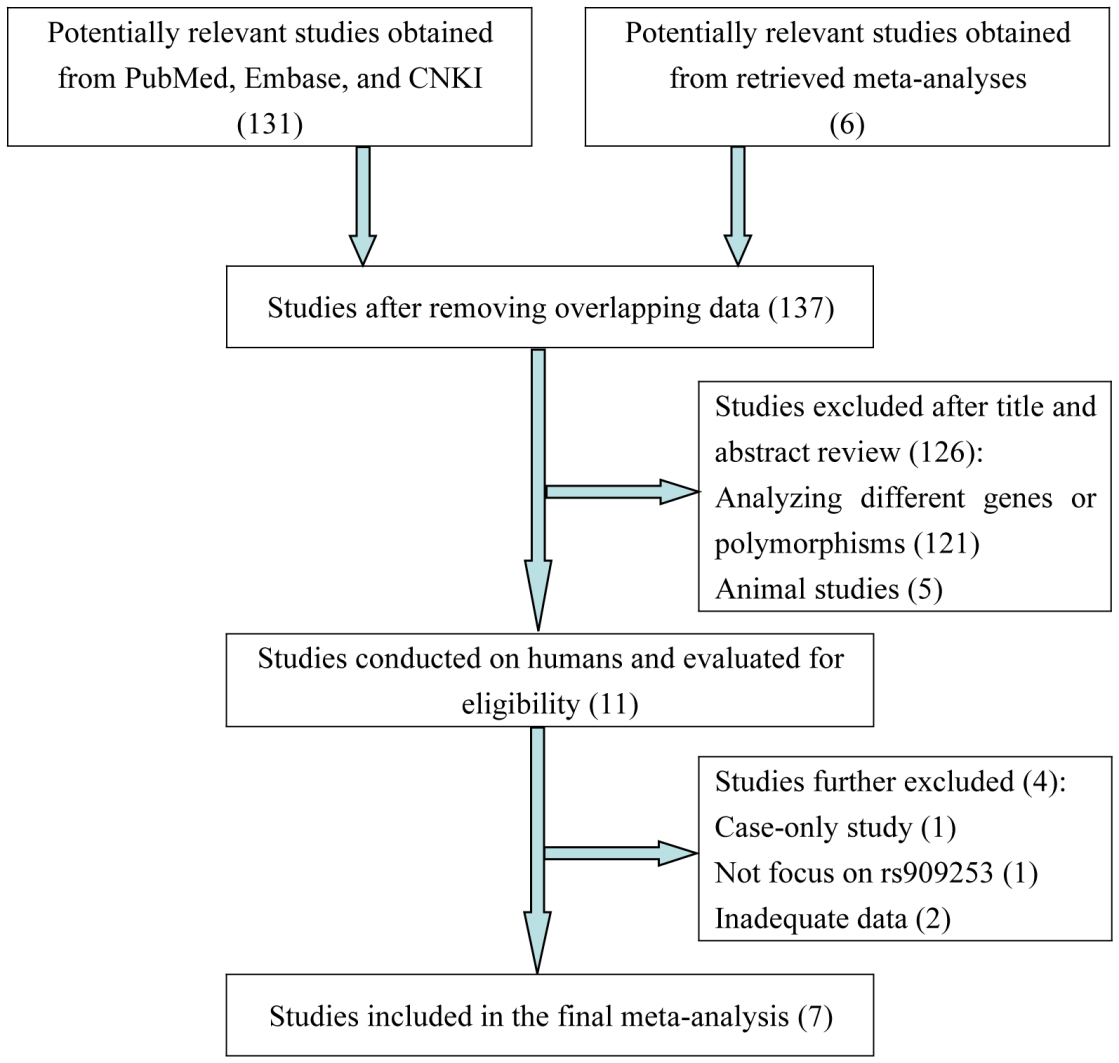
Flowchart depicting literature search and selection.

The systematically electronic along with manual searches supplied 137 papers that may have usable data for this meta-analysis. However, 126 of them were removed after preliminary review of titles and abstracts. Among the remaining 11 papers, we screened the full texts to examine their eligibility based on the inclusion criteria. As a result, 4 studies were excluded (one was carried out without control population; one focused on other polymorphisms rather than the 252A>G polymorphism; two presented insufficient genotype data for risk estimate) [24]–[27] and thus 7 studies [15]–[19], [28], [29] were considered eligible for the present analysis (Figure 1), including four Caucasian studies and three Asian studies. All of the included studies were case-control designed, comprising 5 557 migraineurs and 20 543 unrelated healthy controls. In addition, five studies provided available data for MA and seven for MO. None of the genotype distributions in each control group was significantly deviated from HWE (Table 1 and 2).

**Table 1:** General characteristics of the studies included in the present meta-analysis.

First Author	PublicationYear	No. of cases/controls	MAF (cases/control)	Racial Descent	Study Country	Type of Controls	Mean Age
Trabace	2002	77/101	0.253/0.386	Caucasian	Italy	Unrelated healthy controls	39.7±7.4, 36.7±6.9
Lee	2007	439/382	0.468/0.425	Asian	Korea	Healthy controls	47.8±11.2
Asuni	2009	299/278	0.183/0.133	Caucasian	Italy	Healthy Sardinian controls	35.28±10.22
Schurks	2009	4332/19269	0.337/0.336	Caucasian	USA	NS	NS
Ghosh	2010	216/216	0.241/0.241	Asian	India	Healthy controls	32.08±12.28
Pappa	2010	103/178	0.189/0.216	Caucasian	Greece	Unrelated healthy controls	10.5±0.7
Ishii	2012	91/119	0.390/0.328	Asian	Japan	Non-headache healthy volunteer	42.4±10.2

NS: not specified in original paper.

**Table 2:** Available data for meta-analysis of MA and MO risk in association with TNF-β 252A>G polymorphism.

First Author	Publication Year	MA		MO	
		No. of Cases	No. of Controls	No. of Cases	No. of Controls
Trabace	2002	30	101	47	101
Lee	2007	65	382	327	382
Asuni	2009			299	278
Schurks	2009	1213	19269	1824	19269
Ghosh	2010	84	216	132	216
Pappa	2010			103	178
Ishii	2012	24	119	67	119

MA: migraine with aura, MO: migraine without aura.

### Quantitative synthesis

**Figure 2:**
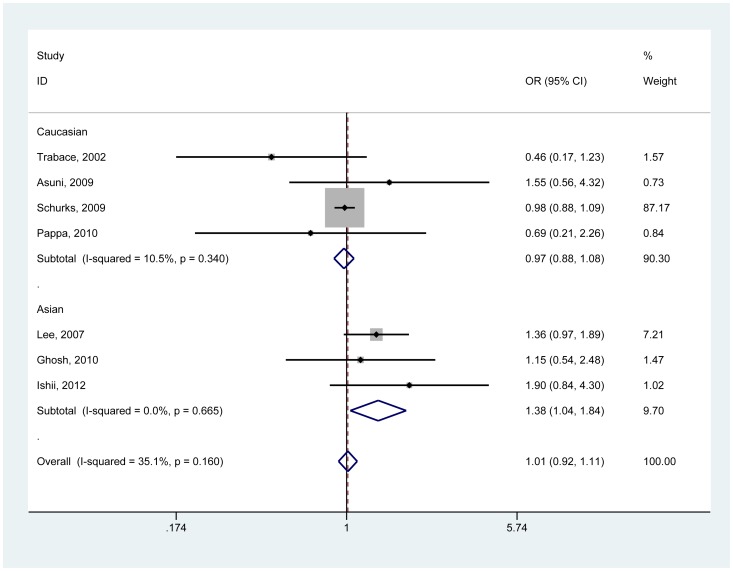
Forest plot of migraine risk associated with *TNF-β* 252A>G polymorphism stratified by ethnicity under GG vs. AG + AA model. The boxes and horizontal lines represent the OR and the corresponding 95% CI. The area of the boxes indicates the weight (inverse of the variance). The diamond correspond to the summary OR and 95% CI.

Table 3 lists the main meta-analysis results calculated with the fixed effects model. By pooling the 7 selected studies, non-significant association between 252A>G polymorphism and migraine was observed under all genetic models. Strikingly, the meta-analysis provided an OR of 1.38 (95%CI, 1.04–1.84; P for heterogeneity, 0.665) for migraine associated with 252A>G polymorphism under the recessive model (GG vs. AG + AA) in Asian populations (Figure 2). None of the contrast models showed a significant association in Caucasian populations.

**Table 3:** Main results of the pooled data in the present meta-analysis.

Subgroups	GG vs. AA		GG + AG vs. AA		GG vs. AG + AA		G vs. A		AG vs. AA	
	OR (95%CI)	P (Q-test)	OR (95%CI)	P (Q-test)	OR (95%CI)	P (Q-test)	OR (95%CI)	P (Q-test)	OR (95%CI)	P (Q-test)
**Total**	1.01 (0.92, 1.12)	0.246	1.01 (0.96, 1.06)	0.538	1.01 (0.92, 1.11)	0.160	1.01 (0.97, 1.06)	0.139	1.01 (0.96, 1.07)	0.658
**Ethnicity**										
Caucasian	0.98 (0.89, 1.09)	0.204	1.01 (0.96, 1.07)	0.179	0.97 (0.88, 1.08)	0.340	1.00 (0.96, 1.05)	0.047	1.02 (0.96, 1.08)	0.259
Asian	1.29 (0.95, 1.75)	0.717	1.03 (0.87, 1.21)	0.944	**1.38 (1.04, 1.84)**	0.665	1.09 (0.95, 1.25)	0.765	0.98 (0.81, 1.19)	0.985
**Subtype**										
MA	1.04 (0.87, 1.24)	0.440	1.02 (0.93, 1.12)	0.952	1.02 (0.87, 1.21)	0.174	1.02 (0.95, 1.10)	0.735	1.03 (0.93, 1.13)	0.887
MO	1.03 (0.90, 1.18)	0.284	1.01 (0.94, 1.08)	0.540	1.05 (0.92, 1.20)	0.248	1.01 (0.95, 1.07)	0.120	1.00 (0.93, 1.08)	0.711
**Gender**										
Migraine	0.76 (0.35, 1.67)	0.569	0.91 (0.65, 1.29)	0.910	0.80 (0.37, 1.72)	0.552	0.90 (0.67, 1.22)	0.679	0.92 (0.64, 1.33)	0.953
MO	0.53 (0.20, 1.36)	0.934	0.89 (0.61, 1.30)	0.971	0.54 (0.22, 1.38)	0.903	0.85 (0.61, 1.19)	0.988	0.92 (0.62, 1.38)	0.933

When stratifying the data by type of migraine, we did not observe either an increased risk or decreased risk of MA or MO. Subgroup analysis based on gender provided similar results that as compared with men, women had no higher risk of developing migraine or MO (Table 3).

**Figure 3:**
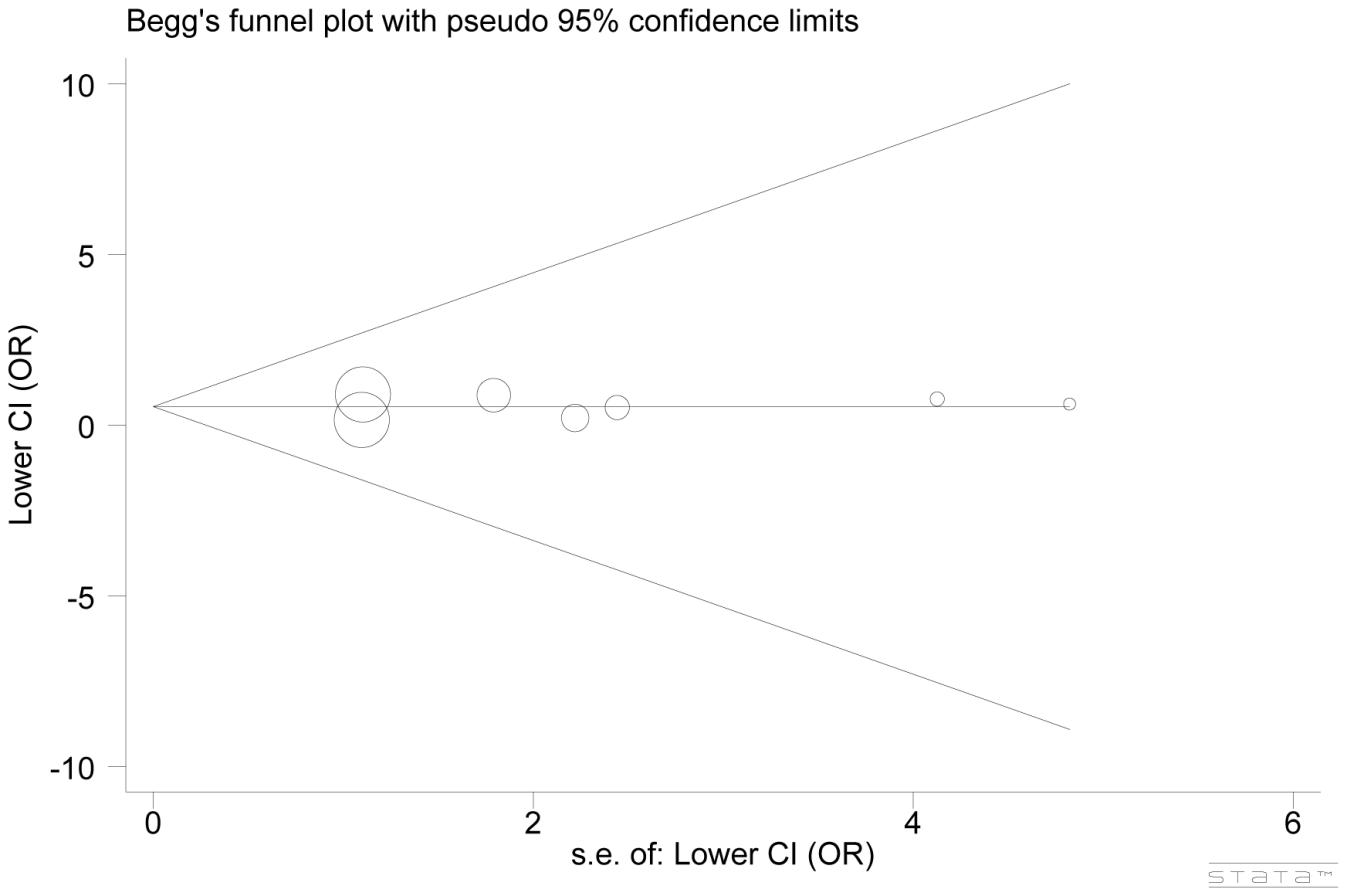
Begg's funnel plot for *TNF-β* 252A>G polymorphism. Log OR is plotted versus standard error of Log OR for each included study. Each circle dot represents a separate study for the indicated association between *TNF-β* 252A>G polymorphism and migraine risk under GG vs. AA model.

To explore the potential sources of heterogeneity on the overall results, we conducted sensitivity analysis and the initial ORs were not significantly influenced by sequential removal of each study from the total analysis. Egger's test and the Begg's funnel plots performed in all contrast models provided no statistical evidence of publication bias (P, 0.900 and P, 1.000, respectively for the two tests for the homozygous model) (Figure 3).

## Discussion

Migraine is a primary headache disorder with its aetiology being incompletely understood. In recent years, a great deal of attention has been directed to the field of migraine genetics [30]. Migraine is recognized as a polygenic disorder induced by a wide range of candidate genes that confer a minor nonetheless significant impact on the susceptibility [31]. Evidence from twin studies showed that approximately 40% of total migraine can be attributable to genetic susceptibility [32], [33]. *TNF* is a cytokine involved in the mediation of inflammatory reactions and endothelial function, and it has been implicated in inflammation-related hyperalgesia [15], [16]. *TNF*-α, a potential pain mediator in neurovascular inflammation, has been reported to increase migraine risk in the population of Italian ancestry [34]. *TNF-β* is another subunit of *TNF* gene and has been frequently investigated in the studies concerning migraine risk.

A previous investigation into the associations between *TNF* gene polymorphisms and migraine presented the discoveries indicating that there was no overall association between the 252A>G polymorphism and migraine. Neither did the investigators find effect modification of MA or MO in women or men in the meta-analysis, where ethnicity-specific analysis was not carried out [35]. Although our meta-analysis including one more new dataset also suggested no significant associations in either women or men with migraine or MO, an OR that suggested statistically increased risk of migraine was observed in Asian populations. This finding may help to expand our understanding of the aetiology mechanism underlying migraine.

In our study, we observed statistical evidence for a significant association of the 252A>G polymorphism with migraine in Asian subjects, but not in Caucasians. Multiple possibilities may result in the differential relation, but there are two most likely explanations. One refers to the study size. Notably, the number of Caucasian subjects is much larger relative to the Asian subjects (24 637 vs. 1 463), which assures an adequate detection power in Caucasian population. And we can not exclude the possibility that the current positive association revealed in Asians may be derived by chance. The other explanation may be the different frequency of the G allele between the two populations: for Asians, the frequency is 37.4% and 22.6% for Caucasians. This implies that the genetic background is a potential factor influencing the genetic predisposition to migraine.

Heterogeneity and publication bias are two inevitable confounding factors which could distort the true associations between SNPs and diseases under research when performing meta-analysis. Considerable attention should be paid in interpreting the findings if the two factors are obviously indicated in related tests. Fortunately, as no significant heterogeneity and publication bias were revealed in our meta-analysis, the results for the association assessed in the present study are relatively convincing by using a rigorous analytic approach. However, the possibility that the effect size of risk estimate is derived by chance can not be excluded, because the total number of cases in general analysis, not to mention the subgroup analyses, is not large enough to obtain a precise assessment of the association between 252A>G polymorphism and migraine. Further, influence from environmental factors is of substantial importance in risk estimate. Such exploration was precluded owing to the inadequate data.

To sum up, these data provided further evidence that 252A>G polymorphism in the TNF region may represent a risk factor for the development of migraine in the population of Asian ancestry. No statistical evidence was indicated in subgroup analyses by type of migraine and gender. Further investigations using a much larger sample with interplay between environment and gene taken into account are clearly needed to validate the findings in this meta-analysis.

## Supporting Information

Checklist S1 **PRISMA Checklist.**
(DOC)
